# Patent arterial duct

**DOI:** 10.1186/1750-1172-4-17

**Published:** 2009-07-10

**Authors:** Jonathan T Forsey, Ola A Elmasry, Robin P Martin

**Affiliations:** 1 Bristol Congenital Heart Centre, Bristol Royal Hospital for Children and Bristol Royal Infirmary, University Hospitals Bristol, NHS Foundation Trust, Bristol, UK; 2 Pediatric Department, Faculty of Medicine, Ain Shams University, Cairo, Egypt

## Abstract

Patent arterial duct (PAD) is a congenital heart abnormality defined as persistent patency in term infants older than three months. Isolated PAD is found in around 1 in 2000 full term infants. A higher prevalence is found in preterm infants, especially those with low birth weight. The female to male ratio is 2:1. Most patients are asymptomatic when the duct is small. With a moderate-to-large duct, a characteristic continuous heart murmur (loudest in the left upper chest or infraclavicular area) is typical. The precordium may be hyperactive and peripheral pulses are bounding with a wide pulse pressure. Tachycardia, exertional dyspnoea, laboured breathing, fatigue or poor growth are common. Large shunts may lead to failure to thrive, recurrent infection of the upper respiratory tract and congestive heart failure. In the majority of cases of PAD there is no identifiable cause. Persistence of the duct is associated with chromosomal aberrations, asphyxia at birth, birth at high altitude and congenital rubella. Occasional cases are associated with specific genetic defects (trisomy 21 and 18, and the Rubinstein-Taybi and CHARGE syndromes). Familial occurrence of PAD is uncommon and the usual mechanism of inheritance is considered to be polygenic with a recurrence risk of 3%. Rare families with isolated PAD have been described in which the mode of inheritance appears to be dominant or recessive. Familial incidence of PAD has also been linked to Char syndrome, familial thoracic aortic aneurysm/dissection associated with patent arterial duct, and familial patent arterial duct and bicuspid aortic valve associated with hand abnormalities. Diagnosis is based on clinical examination and confirmed with transthoracic echocardiography. Assessment of ductal blood flow can be made using colour flow mapping and pulsed wave Doppler. Antenatal diagnosis is not possible, as PAD is a normal structure during antenatal life. Conditions with signs and symptoms of pulmonary overcirculation secondary to a left-to-right shunt must be excluded. Coronary, systemic and pulmonary arteriovenous fistula, peripheral pulmonary stenosis and ventricular septal defect with aortic regurgitation and collateral vessels must be differentiated from PAD on echocardiogram. In preterm infants with symptomatic heart failure secondary to PAD, treatment may be achieved by surgical ligation or with medical therapy blocking prostaglandin synthesis (indomethacin or ibuprofen). Transcatheter closure of the duct is usually indicated in older children. PAD in preterm and low birth weight infants is associated with significant co-morbidity and mortality due to haemodynamic instability. Asymptomatic patients with a small duct have a normal vital prognosis but have a lifetime risk of endocarditis. Patients with moderate-to-large ducts with significant haemodynamic alterations may develop irreversible changes to pulmonary vascularity and pulmonary hypertension.

## Definition and diagnostic criteria

The arterial duct is an essential fetal structure and only becomes abnormal if it remains open after the neonatal period. Persistence of the arterial duct has been defined as continued patency in term infants older than 3 months [1]. It can be defined by combining both the clinical features and anatomical measurements acquired from echocardiogram as small, moderate or large and by configuration on angiogram, as described by Krichenko *et al. *[2].

## Epidemiology

Isolated patent arterial duct is found in around 1 in 2000 full term infants, constituting nearly 10% of all congenital heart disease [3] and is the second most common congenital heart defect [4]. The overall incidence in infants born prematurely is 8 per 1000 live births with extremely high incidence of ductal patency in low birth weight infants [1]. Siassi *et al. *[5] reported that the incidence of patent arterial duct was 21% in a prospective study on 150 preterm infants with low birth weight. However, spontaneous delayed closure of the arterial duct occurred in 79% of their patients who survived the immediate neonatal period. The majority of reports show that there is a higher incidence in females, with a female to male ratio of ~2:1. Studies have revealed recurrence rates of between 1–5% among siblings of individuals with isolated patent arterial duct [6-10]. There are case reports which have shown much higher recurrence rates in individual families, suggesting different inheritance patterns in these families [11]. The familial occurrence of patent arterial duct is uncommon. An epidemiology paper from Carleton covering the period between 1941–58 found only 41 families where the same cardiac defect occurred in two family members and of these only 17 had patent arterial duct [12].

## Clinical description

Most patients are asymptomatic (when the duct is small). In a moderate-to-large duct, the patency of the arterial duct is recognised on detection of the characteristic continuous heart murmur. Often termed a "machinery murmur", this is typically loudest at the left upper chest or infraclavicular area. The precordium may be hyperactive and peripheral pulses are bounding with a wide pulse pressure. The patient may have tachycardia and in older children there may be a history of exertional dyspnoea. There may be history of prematurity, asphyxia, or maternal rubella. The baby may have fast or laboured breathing, become tired easily, or display poor growth. Large shunts may lead to failure to thrive, recurrent infection of the upper respiratory tract and congestive heart failure [1]. There is a subset of patients who have been found to have a patent duct but remain asymptomatic and have no audible murmur, this group is often referred to as having a 'silent' duct.

In premature infants, there should be a high index of suspicion. Often ventilated infants with hyaline membrane disease will improve followed by an inability to wean ventilation or require increased support. There may be episodes of apnoea, bradycardia or general cardiovascular instability. Physical examination may be similar to older children with tachycardia, a continuous murmur and bounding pulses however it is not uncommon for the murmur to be only systolic.

In patients where a moderate-to-large patent duct has not been previously diagnosed, irreversible pulmonary vascular changes may occur secondary to prolonged exposure of high pulmonary flow and pressure. These changes include arteriolar medial hypertrophy, intimal proliferation and fibrosis. In this group of patients when pulmonary arterial pressure exceeds systemic pressure, the flow across the duct is reversed and is right to left. This results in the patient becoming cyanosed which may be differential with cyanosis and possibly clubbing seen in the feet but not the fingers. The murmur may disappear with accentuation of the second heart sound as a result of high pulmonary pressure. These patients will have a progressive reduction in their exercise capacity.

## Aetiology

The arterial duct is the arterial connection between the pulmonary artery and the aorta that shunts blood away from the lungs during fetal life. In normal cardiovascular embryological development the arterial duct is formed from the sixth pair of embryonic arches. The distal portion of the left sixth arch persists as the arterial duct, connecting the left pulmonary artery with the left dorsal aorta with regression of the right distal arch. This process is completed by the 8^th ^week of fetal life. The duct normally constricts shortly after birth due to the postnatal drop in circulating prostaglandin E2 levels, as well as the rise in systemic oxygen tension, which induce a cytochrome P450-dependent increase in endothelin-1 in ductal smooth muscle cells [13]. Subsequent to this constriction, the ductal endothelial cells detach, the subendothelial region swells leading to the formation of subintimal cushions, and the smooth muscle cells migrate to the duct region, resulting in complete ductal closure and formation of the ductal ligament [14].

In a term infant with a patent arterial duct, the structure of the ductal wall is abnormal. Histologically, the internal elastic lamina of the arterial duct is generally intact and the internal cushions are absent or less well formed than usual [15]. In the premature infant, persistent patency of the arterial duct is usually a result of hypoxia or immaturity, with the duct usually having a normal structure [16].

In the majority of cases of patent arterial duct there is no identifiable cause. These cases represent the influence of multifactorial inheritance [17]. Persistence of the duct is associated with chromosomal aberrations, asphyxia at birth, birth at high altitude, and congenital rubella [1]. It has been suggested that there is a higher incidence in children born to mothers with *TT genotype *of *MTHFR C677T *locus involved in homocysteine metabolism [18], although there are environmental influences contributing to these findings.

Occasional cases are associated with specific genetic defects. These include chromosomal defects, such as trisomy 21 and 18, deletion syndromes 4q-, 16p13.13 (Rubinstein-Taybi) and 9p- (CHARGE). Issekutz's epidemiological study looking at infants diagnosed with CHARGE syndrome in Canada demonstrated a higher incidence of patent arterial duct in subgroup B, who present with only three or fewer major criteria [19]. Familial patent arterial duct can be either isolated or part of a syndrome.

### Isolated familial patent arterial duct

#### • Autosomal dominant inheritance

Families with a high incidence of persistent patency of the arterial duct have been reported by several researchers [11,20-23]. In these families, the incidence of patent arterial duct is higher than might be expected with a polygenic mode of inheritance. In one report, there was a high frequency of patent arterial ducts in three generations affecting both sexes in the absence of consanguinity, highly suggestive of an autosomal dominant mode of inheritance [11].

#### • Autosomal recessive inheritance

Wei *et al. *described a family in which four out of six children of the same family were affected with patent arterial duct [24]. Their parents and grandparents were all free of congenital heart disease. They suggested that autosomal recessive inheritance might play a role, as other potential causes (*e.g. *maternal rubella, maternal alcohol abuse, and high altitude) could be excluded from this case.

Mani *et al. *found that patent arterial duct accounted for 15% of congenital heart disease in Iran, compared to 2%–7% in the United States [25]. Moreover, parental consanguinity was significantly higher among patients with patent arterial duct (63%) than among the general Iranian population (25%) or among control cases with tetralogy of Fallot (30%). The recurrence of patent arterial duct among siblings was 5%. A genome-wide analysis of linkage in 21 unrelated consanguineous patent arterial duct cases demonstrated a multipoint logarithm, with an odds score of 6.27 in favour of linkage of patent arterial duct to a 3-centimorgan interval of chromosome 12q24 with 53% of kindred's linked. Together, these findings suggest that there is a recessive component involved in patent arterial duct and implicate a single locus in one third or more of all patent arterial duct cases in Iran. They further suggest a role for this locus in patent arterial duct worldwide. The authors suggest that persistent patency of the arterial duct is commonly a recessive disease with incomplete penetrance.

### Familial patent arterial duct as part of a syndrome

#### • Char syndrome

Char syndrome is an autosomal dominant condition comprising ptosis, low-set ears, short philtrum and 'duck-bill' lips, patent arterial duct, a single fifth finger flexion crease with two fifth finger phalanges and mild learning difficulties. Other features described include a broad forehead, flared eyebrows, mild hypertelorism, flat midface and strabismus. First described by Char in 1978, this syndrome has subsequently been reported by several subsequent investigators [26-29]. The facial findings were less pronounced in older relatives and a change in phenotype with age has been reported by different researchers [26,28]. Additionally, the phenotype has been reported to vary, even within families, presumably due to different mutations in the same gene. The patent arterial duct in five multigenerational families studied has been of variable penetrance.

Char syndrome has been mapped to a narrow region of chromosome 6p12-p21 [29]. Further studies have shown that Char syndrome is caused by defects in *TFAP2B*, encoding a neural crest-related transcription factor (also known as AP-2β). The *TFAP2 *genes play an important role in retinoic acid-induced differentiation, particularly in cells derived from the neuroectoderm. Thus, it appears that the patent arterial duct in Char syndrome is likely a result of abnormal neural crest development [30].

#### • Familial thoracic aortic aneurysm/dissection (TAA/AD) with patent arterial duct

Khau Van Kien *et al. *described a single large three-generational French family with a vascular syndrome that associates TAA/AD with patent arterial duct [31]. Genetic analysis of this family identified a locus at chromosome 16p12.2-p13.13 with aortic stiffness as an early hallmark of the disease [32]. Mutations in the *MYH11 *gene were eventually identified, which encodes the smooth muscle myosin heavy chains, however this is only thought to be involved in rare instances in sporadic instances of patent duct [33]. The mode of inheritance appeared to be autosomal dominant and genetic linkage analysis excluded involvement of the previously described loci associated with syndromic and non-syndromic familial TAA/AD, Char syndrome and recessive patent arterial duct. Similar suggestions were also made by Glancy *et al. *and Telen *et al. *[34,35]. The former also described a single family in whom TAA/AD and patent arterial duct occurred in three generations with apparent autosomal dominant inheritance. In all these studies no other features of typical connective disorders were observed.

#### • Familial patent arterial duct and bicuspid aortic valve with hand anomalies

A novel inherited syndrome has recently been described comprising variable cardiovascular defects (patent arterial duct, bicuspid aortic valve, pseudocoarctation of the aorta), hand anomalies (consisting of 5th metacarpal hypoplasia of varying degree), and generalised brachydactyly. Linkage analysis with this moderate-sized kindred using polymorphic DNA markers from the Char syndrome locus excluded the possibility that this family was inheriting an allelic variant of Char syndrome. Inspection of the pedigree favoured inheritance by a dominant mechanism. However, X-linked dominance could not be excluded. Penetrance appeared to be complete, but there was variability in the cardiac and hand phenotypes [36].

## Diagnostic methods

**Figure 1:**
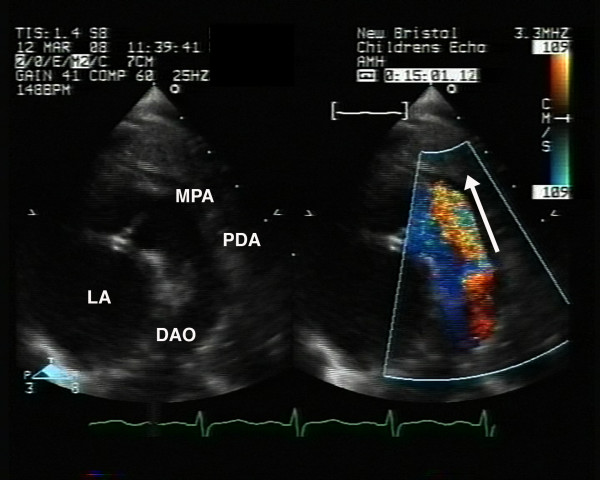
**Echocardiography, a high parasternal long axis view demonstrating a large patent duct (PDA)**. Colour flow demonstrates shunting from 'left to right', from the descending aorta (DAO) to the pulmonary arteries (MPA).

Diagnosis is usually possible based on clinical examination. The confirmation of a patent arterial duct is made with transthoracic echocardiography. In the majority of cases, cross sectional echocardiography can accurately define the presence and characteristics of a patent arterial duct. The most commonly employed views are left parasternal, high left parasternal, suprasternal and short axis views [37]. The arterial duct can be measured on standard two dimensional views in the high parasternal position where it forms a 'three-legged' appearance with the pulmonary arteries. Assessment of ductal blood flow can be made using colour flow mapping (Figure 1) and pulsed wave Doppler. Particular attention should be paid to the direction and flow pattern to gain information relating to restrictive flow pattern, shunt assessment and an indirect indication of pulmonary arterial pressure. However, it may be difficult to image the ductus in older patients, transoesophageal echocardiography with colour flow mapping has shown increased sensitivity in adolescent and adult patients [38,39]. As pulmonary vascular resistance falls after birth, the left to right shunt increases, leading to increased pulmonary over circulation and increased pulmonary venous return. As a consequence, there is left atrial and left ventricular volume loading, seen as enlargement of the left atrium and ventricle on apical four chamber and long axis views. As a result of ventricular enlargement, there may be a degree of mitral regurgitation. The degree of volume loading can be approximated using the left atrium:aortic root ratio, a significant shunt being considered one that gives a value of > 1.3 [37,40]. This is best assessed using the m-mode in a standard long axis view.

**Figure 2:**
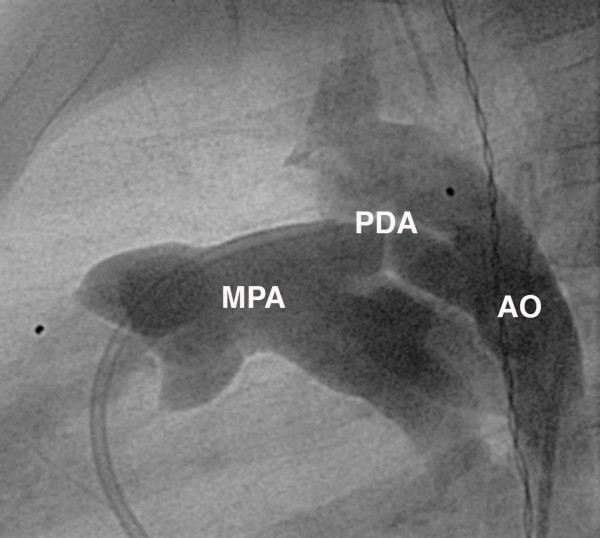
**Lateral angiogram demonstrating a large tubular type duct (type C) with aortic (AO) to pulmonary flow (MPA)**. The catheter can be seen entering the MPA from the right ventricle, a second pigtail catheter is positioned in the descending aorta from which contrast is delivered.

**Figure 3:**
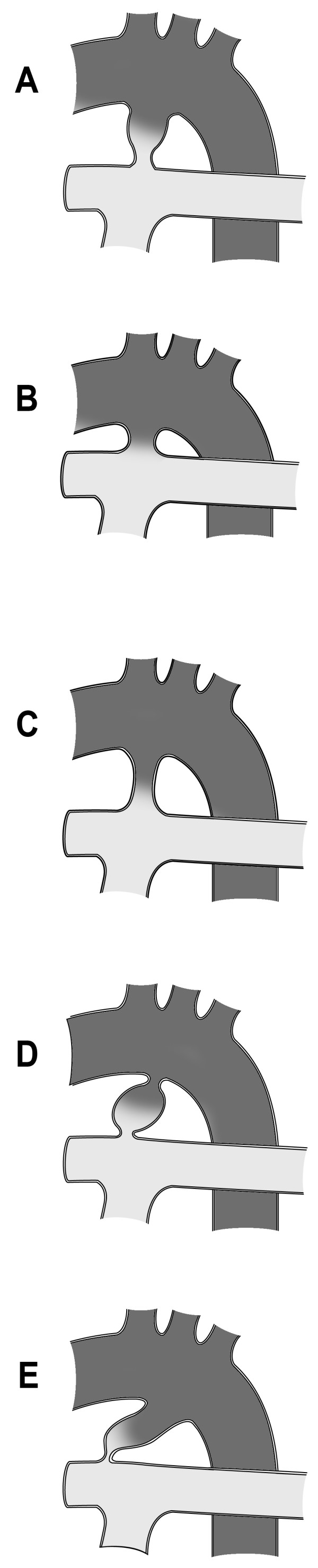
**Diagrammatic representation of the configuration of the ductus as demonstrated on the lateral angiogram can be classified as described by Krichenko**. Type A or conical duct with well defined aortic ampulla and constriction near the pulmonary artery end. Type B is a large duct with window like structure which is very short in length. Type C is a tubular duct without any constriction. Type D is the complex duct with multiple constrictions and the type E or elongated duct with constriction remote from the edge of the trachea as viewed on lateral angiography (figure adapted from Amplatzer™ manufacturer information).

Diagnostic cardiac catheterisation with angiography is rarely necessitated in patients with typical clinical and echocardiographic findings. Predominately catheterisation is limited to transcatheter therapies for occlusion of the duct. In this setting, ductal anatomy is usually defined with aortography (Figure 2). The configuration of the ductus as demonstrated on the lateral angiogram can be classified as described by Krichenko *et al. *[2]. The Type A or conical duct has a well defined aortic ampulla and constriction near the pulmonary artery end. Type B is a large duct with window-like structure, which is very short in length. Type C is a tubular duct without any constriction. Type D is the complex duct with multiple constrictions and the type E or elongated duct with constriction remote from the edge of the trachea as viewed on lateral angiography (Figure 3). This classification system allows the interventionalist to assess the size and configuration of the duct and select the most suitable occlusion device.

## Genetic counselling

There have been significant advances in the understanding and identification of genetic causes of congenital heart disease in the last decade and continues to be a field which is changing rapidly [41]. The recognition of subgroups of patients with a stronger genetic component to the aetiology of patent arterial duct is important for the counselling of couples with concerns regarding the risks of recurrence. The recognition of familial causes of patent arterial duct stresses the importance of a full family history and of careful examination of the facial features before estimating recurrence risk for patent arterial duct.

## Antenatal diagnosis

Antenatal diagnosis of patent arterial duct is not possible as it is a normal structure during antenatal life. At present, there are no antenatal genetic investigations that can reliably predict the patency of the arterial duct in postnatal life.

## Differential diagnosis

Conditions in which there are signs and symptoms of pulmonary over circulation secondary to a left to right shunt must be excluded. Other conditions may also present with a continuous murmur and bounding pulses and differentiation from an arterial duct must be made on echocardiogram. These include: coronary, systemic and pulmonary arteriovenous fistula, collateral vessels, peripheral pulmonary stenosis and ventricular septal defect with aortic regurgitation [42].

## Management

Preterm infants with symptomatic heart failure secondary to persistent patency of the arterial duct may be treated by surgical ligation or medically with conservative management and indomethacin or ibuprofen. Medical intervention is usually the treatment of choice due to the risks involved with surgical ligation. Initial conservative management involves fluid restriction and diuretic therapy with optimisation of calorie intake and ventilator support. These actions will not aid ductal closure but can improve respiratory physiology and symptoms in the short term. Indomethacin and ibuprofen act as inhibitors of prostaglandin forming cyclo-oxygenase (COX) enzymes. No statistical difference has been demonstrated between ibuprofen and indomethacin in their effectiveness in closing a patent duct [43]. Indomethacin has greater inhibition of COX-1 receptors and consequently greater vasoconstrictive effects with ibuprofen offering relatively less inhibition. Both drugs have potentially serious adverse effects with indomethacin associated with renal dysfunction, necrotising enterocolitis and impairment of cerebral blood flow but potentially protective effect against intraventricular haemorrhage [44,45]. It has been suggested that ibuprofen may increase the risk of chronic lung disease and pulmonary hypertension has been described when ibuprofen was given prophylactically [46]. Both drugs are protein bound and so in the neonate can potentially compete with bilirubin for albumin binding sites, resulting in an increase in free bilirubin and the risk of bilirubin encephalopathy [47]. Timing of intervention and dosage of pharmacologic treatment remains a debated topic. Indomethacin can be given as either a short course of three doses 12 hourly or as a long course of six doses 24 hourly. There is no difference in ductal closure rates between the two but the longer course has a lower incidence of ductal reopening [48,49]. It is known that the optimal time for treatment is less than a week of age, however this must be balanced against treating infants and exposing them to potential side effects when the duct would have closed spontaneously. Current trends would support treatment within the first week of life and in symptomatic older infants but accepting that treatment failure and ductal reopening may well occur in the older age infants [50].

A number of infants will either not be suitable or fail medical management. These include infants with renal impairment, gastrointestinal pathology including previous necrotising enterocolitis and patients with platelet dysfunction. In such cases, surgical division and ligation is indicated for closure of a significant arterial duct. This is performed via a thoracotomy and whilst high closure rates and low mortality are reported there may be significant morbidity. These include bleeding, pneumothorax, chylothorax, infection, haemodynamic instability and thoracic scoliosis. A minimally invasive surgical approach employing video-assisted thoracic surgery (VATS) has shown early results comparable to thoracotomy including premature and low birth weight infants [51,52].

**Figure 4:**
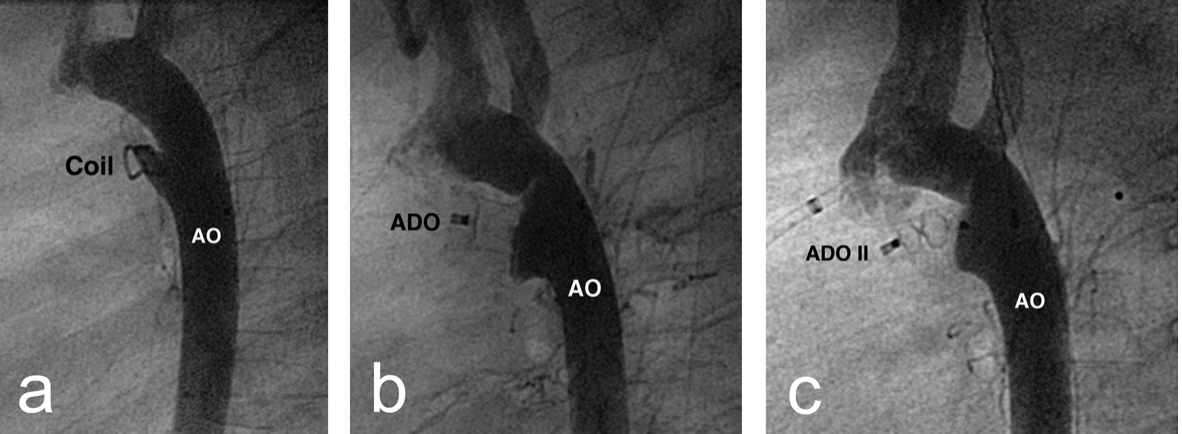
**a) Lateral angiogram showing a Cook™ flipper coil with complete occlusion of the duct on angiography; b) The same patient as demonstrated in figure 2 that has been successfully closed using an Amplatzer ductal occluder™ (ADO); c) Successful closure of the patent duct demonstrated in figure 1 with an Amplatzer ductal occluder II ™ (ADO II)**.

**Figure 5:**
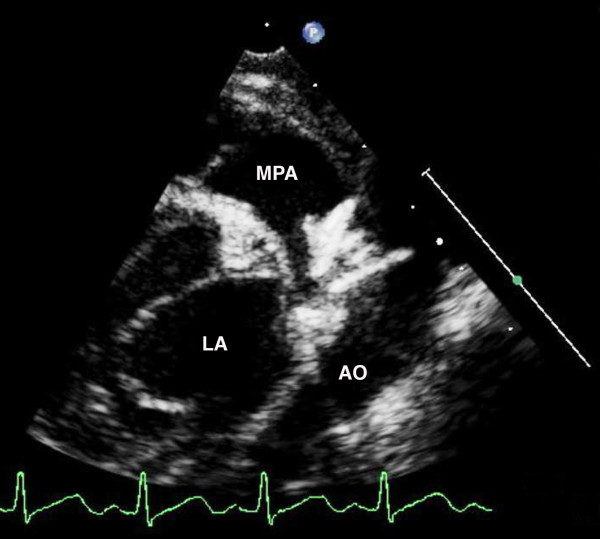
**Echocardiogram following closure of patent duct with an Amplatzer ductal occluder II™**. This image is taken from the same patient as figure 3c. This high parasternal long axis clearly shows the device fixed in the duct between left pulmonary artery and descending aorta.

In children outside of this age group, the management decisions are based on the control of symptomatic pulmonary over circulation and the lifetime risk of endocarditis. Transcatheter closure of the duct is usually offered to all children outside of the neonatal period. There have been several studies reporting excellent results with low complication rates for a variety of occluders (Figure 4a–c, 5) [53-56]. Surgical ligation remains an option for those unsuitable for transcatheter closure. This is now rare with the range of occluders developed but may include very large ducts where adequate anchorage of the occluder is not possible.

The best management for the 'silent' duct remains debatable with many centres electing not to close either surgically or via catheter techniques. The risk of endocarditis is low, although studies have reported an increase in the incidence in these patients [57]. This has prompted some centres to offer elective closure of the patent duct to all patients [58].

## Prognosis

The prognosis for the untreated patent arterial duct depends largely on the size of the duct and the degree of left to right shunt and pulmonary over circulation. In preterm and low birth weight infants a patent duct is associated with significant co-morbidity and mortality due to haemodynamic instability. These include pulmonary oedema and haemorrhage, bronchopulmonary dysplasia, intraventricular haemorrhage necrotizing enterocolitis as well as congestive cardiac failure. Patients with a small duct may exhibit no symptoms and have no haemodynamic impairment, these patients have a normal prognosis but do have a lifetime risk of endocarditis. Those patients with moderate-to-large ducts with significant haemodynamic changes may present with evidence of congestive cardiac failure or in the long term may develop irreversible changes to pulmonary vascularity and pulmonary hypertension [59]. Patients presenting with signs of cardiac failure would usually be offered either transcatheter or surgical closure of the duct. Irreversible pulmonary vascular changes or Eisenmenger's syndrome as a result of duct is fortunately rare, but in such cases treatment is largely supportive.

For patients who undergo transcatheter closure the immediate occlusion rates are in excess of 90% and immediate complication rates are low [53-56,60,61]. There is potential for left pulmonary artery and descending aortic obstruction in patients who are of low weight with a large duct requiring a relatively large occluder for closure. However, this is rare and normally resolves with age as vessel calibre naturally increases.

Surgical ligation and division of the arterial duct has been shown to have excellent results although is more invasive than transcatheter techniques. It remains an important treatment option for arterial ducts which are unsuitable for transcatheter closure and in premature infants in which medical management has failed. Occlusion rates are in excess of 95% with mortality figures 1–2% [62]. Once the patent duct has been occluded, the long term risk of endocarditis returns to levels for the overall normal population risk. Haemodynamic changes are removed with resolution of a normal circulation.

## Competing interests

The authors declare that they have no competing interests.

## Authors' contributions

JF wrote the first draft of the manuscript. All authors revised the manuscript, read and approved the final manuscript.
